# Human Nasal Epithelial Organoids for Therapeutic Development in Cystic Fibrosis

**DOI:** 10.3390/genes11060603

**Published:** 2020-05-29

**Authors:** Zhongyu Liu, Justin D. Anderson, Lily Deng, Stephen Mackay, Johnathan Bailey, Latona Kersh, Steven M. Rowe, Jennifer S. Guimbellot

**Affiliations:** 1 Gregory Fleming James Cystic Fibrosis Research Center, University of Alabama at Birmingham (UAB), Birmingham, AL 35294, USA; ZLiu@peds.uab.edu (Z.L.); jdanderson@peds.uab.edu (J.D.A.); MackaySS@EVMS.edu (S.M.); lkersh@peds.uab.edu (L.K.); srowe@uabmc.edu (S.M.R.); 2 Department of Pediatrics, Division of Pulmonary and Sleep Medicine, UAB, Birmingham, AL 35233, USA; ldeng@uab.edu (L.D.); johnathb@uab.edu (J.B.); 3 Department of Medicine, Division of Pulmonary, Allergy, and Critical Care Medicine, UAB, Birmingham, AL 35294, USA; 4 Department of Cell, Developmental and Integrative Biology, UAB, Birmingham, AL 35294, USA

**Keywords:** cystic fibrosis, CFTR, human nasal epithelial cells, organoids, biomarker, functional assay, CFTR modulators, pre-clinical in vitro models

## Abstract

We describe a human nasal epithelial (HNE) organoid model derived directly from patient samples that is well-differentiated and recapitulates the airway epithelium, including the expression of cilia, mucins, tight junctions, the cystic fibrosis transmembrane conductance regulator (CFTR), and ionocytes. This model requires few cells compared to airway epithelial monolayer cultures, with multiple outcome measurements depending on the application. A novel feature of the model is the predictive capacity of lumen formation, a marker of baseline CFTR function that correlates with short-circuit current activation of CFTR in monolayers and discriminates the cystic fibrosis (CF) phenotype from non-CF. Our HNE organoid model is amenable to automated measurements of forskolin-induced swelling (FIS), which distinguishes levels of CFTR activity. While the apical side is not easily accessible, RNA- and DNA-based therapies intended for systemic administration could be evaluated in vitro, or it could be used as an ex vivo biomarker of successful repair of a mutant gene. In conclusion, this highly differentiated airway epithelial model could serve as a surrogate biomarker to assess correction of the mutant gene in CF or other diseases, recapitulating the phenotypic and genotypic diversity of the population.

## 1. Introduction

Cystic fibrosis (CF) is an autosomal recessive genetic disorder caused by disease-causing variants in the CF transmembrane conductance regulator (CFTR) gene. CFTR is expressed on the epithelial cells of several organs, including the lung, intestine, pancreas, and liver. In the lung, functional CFTR regulates ion transfer in the airway lumen, balancing the salt, pH, and fluid content as well as influencing the viscosity and organization of mucus. In CF, this balance is impaired, resulting in thick secretions poorly transported out of the lung by the mucociliary apparatus, leading to a cycle of inflammation and infection that ultimately causes significant morbidity and mortality among the CF population.

Identifying an effective cure for CF is critical. While new drugs called CFTR modulators have been identified that are highly effective for the treatment of CF [1,2,3], they target the protein defect in CFTR but do not permanently correct the genetic mutation. Furthermore, although a recent three-drug CFTR modulator therapy was approved for ~90% of patients as defined by the genotype [4,5], no treatment for the underlying defect exists for the remaining 10%. This includes patients with rare mutations unsuccessfully rescued by modulators. Ideally, correction of the mutation using gene therapy or other nucleotide-based approaches would be permanently curative.

The availability of in vitro models to test correction of the genetic defect in CF has been somewhat limited. In this study, we describe the development of an in vitro model derived from patient samples (nasal brush biopsies), which recapitulates the highly differentiated airway epithelium. While we and others reported similar models derived from nasal tissue, some [6,7,8,9] are grown from differentiated cells already in an intact monolayer, either from excised nasal polyps or brush biopsies. In these cases, the apical membrane is to the exterior of the structure, and the basolateral side is not accessible. These spheroids are free-floating, which makes imaging difficult, are of limited quantity, and are derived from cells that are not passaged or expanded, making higher throughput applications impossible. One similar model [10] employs a labor-intensive technical approach that is not amenable to high-throughput applications, which the authors cite as a specific limitation. In contrast, our organoid assay utilizes small amounts of biopsy material that are expanded to millions of cells, which can provide sufficient replicates for moderate-to-high-throughput applications; was optimized for uniformity in organoid distribution and morphology to aid in automated imaging and analysis; and has a greater capacity to detect subtle differences. The model has several potential outcome measures, including quantitating the kinetics of CFTR rescue on fluid transport; fluorescent microscopy to qualitatively determine gene expression; and evaluating the functional activities through the use of novel imaging techniques (micro-optical coherence tomography, µOCT). These are useful to assess the impact of any curative therapy targeted to CFTR mutations [11,12,13,14,15,16]. Because this model is derived directly from patient samples, we postulate that it will reproduce the phenotypic and genotypic diversity that exists among the CF population and will be more likely to predict the effectiveness of the therapy across all patients.

## 2. Materials and Methods

### 2.1. Patient Samples

Non-CF healthy control subjects (n = 12, age 16–40 years) and subjects with CF (n = 36, age 1–51 years) were recruited to this study after written informed consent was obtained. For a subset of subjects with CF who contributed cells for functional assessment, basic descriptive data regarding their age, genotype, pancreatic sufficiency status, and baseline sweat chloride (when available) is described in the Supplementary Material S1. The study was approved by the University of Alabama Institutional Review Board under protocol number IRB-151030001. One brush biopsy from each nare was obtained using cytology brushes (Medical Packaging CYB1, Cat# CYB-1, length: 8 inches, width approximately 7 mm, Camarillo, CA, USA) under direct visualization of the inferior turbinate via an otoscope and sterile 9-mm speculum. The cell number obtained from brushing was varied between subjects, ranging from 2.5 × 10^5^ to 2 × 10^6^.

### 2.2. Cell Culture and Expansion

Expansion of primary human nasal epithelial cells (HNEs) was adapted from conditional reprogramming culture (CRC) methods that have been previously published [17,18]. In brief, after collection, cells were immediately placed in Roswell Park Memorial Institute (RPMI) 1640 media (Thermo Scientific, Waltham, MA, USA) and processed within two hours. Depending on the number of cells collected, cells were either cryopreserved and/or expanded in co-culture with irradiated 3T3 fibroblasts for up to 14 days in a T75 flask containing F-media with 10 μM Y27632 (Stemgent Stemolecule, Beltsville, MD, USA) [17]. At a seeding density of at least 2.5 × 10^3^/cm^2^, one T75 will yield 2 × 10^6^ to 4 × 10^6^ cells. For the initial three days of expansion culture only, Amphotericin B 2.5 µg/mL (Sigma-Aldrich Corp., St. Louis, MO, USA), Tobramycin 100 µg/mL (Alfa Aesar, Ward Hill, MA, USA), Ceftazidime 100 µg/mL (Sigma-Aldrich Corp., St. Louis, MO, USA), and Vancomycin 100 µg/mL (Alfa Aesar, Ward Hill, MA, USA) were added to the media of the CF-derived cells [10]. Cells were discarded if less than 80% confluent by day 14. HNEs were recovered after trypsinization in the expansion flask (0.05% trypsin-EDTA (ethylenediaminetetraacetic acid)). No cells greater than passage 3 were used to avoid a known reduction in CFTR expression at high passage numbers [19]. CF and non-CF cells behaved similarly during expansion, consistent with prior reports [20,21,22].

### 2.3. Differentiated HNE Culture

Organoids were cultured either in a Transwell system (polyester membrane, area 0.33 cm^2^, pore size: 0.4 μm; Corning Inc., Corning, NY, USA) or 15-well angiogenesis slides (ibidi USA, Inc., Fitchburg, WI, USA). Slides were selected to enhance the optical imaging of organoids for morphology and functional assessment. For angiogenesis slides, Matrigel (Corning Inc., Corning, NY, USA) with a total protein concentration of at least 9 mg/mL was used for organoid culture. Each well of the slides was coated with 5 µL of cold 100% Matrigel on ice and slides were placed into a cell culture incubator at 37 °C for at least 30 min. HNEs were resuspended into a 20% matrigel and Ultroser-G (USG) media [23] suspension at 500 cells/µL. For each replicate well, 5 µL of the suspension (2500 cells/replicate) were seeded into imaging slide wells coated with matrigel, incubated at 37 °C for one hour, and covered with 50 µL of USG media. The media was exchanged every other day until the cells were used for experiments. For organoids grown in a Transwell system, a cold Transwell insert was coated with 100 µL of 100% Matrigel on ice, then put into a cell culture incubator at 37 °C for at least 30 min. Sixty microliters of the HNE cell suspension described above were seeded into Transwells coated with matrigel, incubated at 37 °C for one hour, then USG media was added to the lower chamber. For monolayer cultures, Transwell inserts were coated with 0.06 mg/mL in 0.2% acetic acid of human placenta collagen type IV (Sigma-Aldrich Corp., St. Louis, MO, USA). After expansion, HNEs were seeded into the Transwells with the density of 5 × 10^5^ cells/insert in F-media for two days. USG media was exchanged every other day until the cells were well differentiated and used for the short-circuit current (Isc) experiments, around 28 days in culture [24].

### 2.4. Organoid Fixation and Immunoflurescence

Organoids derived from cells donated by 11 unique individals (6 with CF) were harvested after 28 days of culture by replacing media with 50 µL of cold 1X phosphate buffered saline (PBS) in each well on ice and pipetting 3–5 times. For histology and immunofluorescence of cross-sectioned organoids, all dissociated organoids from one ibidi slide (15 wells) were combined into a conical tube on ice. Total volume was adjusted to 10 mL with cold 1XPBS and centrifuged at 4 °C, at 300× *g* for 5 min, and supernatant was removed. Then, 60 µL of warm Histogel (Thermo Scientific, Waltham, MA, USA) was mixed with the organoid pellet, and immediately transferred to a histology mold. Once solid, the mold block was fixed with 4% paraformaldehyde overnight at 4 °C. After embedding in paraffin, the block was then cut into 5-μm cross-sections, fixed onto glass slides, and stained using hematoxylin and eosin (H&E). Some cross-sections were used for immunofluorescence with details described below. Histology was imaged by a Nikon Ts2 microscope. For whole mount immunofluorescence, organoids from one to two wells were pipetted into an eight-well glass bottom chamber slide (ibidi USA, Inc., Fitchburg, WI, USA), which was pre-treated with Cell-Tak (Corning Inc., Corning, NY, USA), removing excess liquid by pipette. The chamber slide was placed into a 37 °C incubator for 40 min to enhance organoid adherence to the glass bottom. After gently washing with 1X PBS 3 times, organoids were fixed with 4% paraformaldehyde (Electron Microscopy Sciences, Hatfield, PA, USA) for 30 min at room temperature (RT), and washed and stored in PBS until immunostaining. Immunofluorescent staining used modifications of previous methods [25,26,27,28]. Briefly, to reduce auto-fluorescence, 250 µL of 50 mM NH_4_Cl in 1X PBS were added into each well of the slides at RT for 30 min while gently shaking. After washing with 1X PBS twice, cultures were permeabilized by 0.1% Triton X-100 (Alfa Aesar, Ward Hill, MA, USA) for 30 min at RT and then blocked with 2% BSA (Thermo Scientific, Waltham, MA, USA) plus 0.1% Triton X-100 in PBS for one hour at RT. All antibody solutions were prepared with 2% BSA plus 0.1% Triton X-100 in PBS. Cultures were incubated with primary antibodies at 4 °C for 2 days as follows: Anti-human CFTR (R&D Systems, Inc., Minneapolis, MN, R domain, MAB1660; 1:100), anti-human ZO-1 (Zona occludens 1; Thermo Scientific, Waltham, MA, USA, MA3-39100-A647; 1:1000), anti-human MUC5B (Mucin 5b; Sigma-Aldrich Corp., St. Louis, MO, USA, HPA008246; 1:100), anti-β IV tubulin (Tubulin β type IV; Abcam, Cambridge, MA, USA, ab11315; 1:100) for cilia, and anti-FOXI1 for Ionocytes (Forkhead box I1; Sigma-Aldrich Corp., St. Louis, MO, USA, HPA071469; 1:100). Cross-sections were incubated with primary antibodies at 4 °C overnight as follows: Anti-human MUC5AC (Mucin 5AC; Thermo Fisher Scientific, Waltham, MA, USA, MA512178; 1:100) for mucin and anti-acetylated tubulin (Tubulin α-4A; Sigma-Aldrich Corp., St. Louis, MO, USA, T7451; 1:100) for cilia. After thoroughly washing with PBS plus 0.3% Triton X-100 three times, 5 min for each time while shaking, all secondary antibodies from Invitrogen were diluted at 1:2000 and incubated at 4 °C for 2 days, except for cross-sections, which were incubated at RT in the dark for one hour. After incubation, the slides were washed thoroughly with PBS with 0.3% Triton X-100 and NucBlue (2 drops/mL for 30 min; 4′, 6-diamidino-2-phenylindole (DAPI); Thermo Scientific, Waltham, MA, USA) in 2% BSA plus 0.3% Triton X-100 was utilized for nuclear staining. Organoids were imaged with either a Nikon Ts2 or confocal microscope (Nikon A1R-HD25).

### 2.5. Imaging and Analysis of Organoids

Organoids were also imaged by either the automated image system in Biotek Lionheart FX or micro-optical coherence tomography (µOCT) [15] in an environmentally controlled chamber at 37 °C and 5% CO_2_. Gen5 ImagePrime software (BioTek, Winooski, VT, USA) in the Lionheart was used for image processing and automated quantitation of the organoid size and count in each well. The forskolin-induced swelling (FIS) assay was adapted from assays described previously [9,29]. FIS assays were performed by 21 days of culture. The organoids for the FIS assay were pre-incubated with NucBlu (Thermo Scientific, Waltham, MA, USA) for 1 h prior to stimulation and imaging. All treatment conditions were diluted in Dulbecco’s PBS and added to media at a 1:1 ratio. The organoids were stimulated with a cocktail of forskolin 10 µM and IBMX 100 µM (FI). Brightfield and fluorescent (DAPI) images of the organoids were then taken in each well of a slide (every 20 min, for 8 h in total) using Lionheart FX. The total surface area (TSA) from the sum of all organoids in the well was automatically determined by the software for each condition at each time point. As an additional quality control, DAPI images were compared to the brightfield to ensure accurate masking of TSA. The change in TSA relative to the baseline (time 0), or fractional change, was calculated for each well (3–5 individual wells per condition) and averaged for each time point. For calculation of the baseline luminal ratio (BLR), brightfield images of 3–5 wells per subject were masked using the polygon function of the NIS-Elements Basic Research software (Nikon Instruments Inc., Melville, NY, USA) by outlining the whole organoid and another mask outlining the lumen of the same organoid. Typically, 40 organoids were analyzed per subject between 3–5 wells, with a minimum of 10 organoids per well. Organoids with lumens that were not able to be visualized and masked accurately were excluded from the analysis. This method was chosen to avoid overestimating the severity of CFTR dysfunction for each subject by assigning these an arbitrarily chosen small area. For µOCT imaging, organoid cultures were placed in an environmentally controlled chamber, images were constructed, and the ciliary beat frequency (CBF) was determined as described previously [15,30,31].

### 2.6. Short-Circuit Current Measurements of Monolayers

Electrophysiological experiments to measure the transport of ions across epithelial monolayers were accomplished using established methods [32]. Reagents were introduced into bath solution in the Ussing chamber (Physiologic Instruments, San Diego, CA, USA) in the following order: Amiloride, 100 μM (apical) to inhibit the ENaC current; forskolin, 10 µM (apical and basal) to stimulate CFTR; VX-770 10 µM (apical and basal) to further potentiate the current; and CFTRinh-172 10 µM (apical). Electrophysiological data were collected and analyzed using Acquire and Analyze 2.3 software (Physiologic Instruments, San Diego, CA, USA) [32].

### 2.7. Statistical Analysis

One-way ANOVA was used for baseline lumen ratio comparisons. Two-way ANOVA with Tukey’s multiple comparisons test was used to evaluate differences between the average fractional change among people with a different CFTR function at 1 and at 8 h. Pearson’s correlation coefficient was calculated for the correlation analysis. Fisher’s exact test was used for culture success comparison between CF and non-CF. All statistical analysis was performed in GraphPad Prism 8 (GraphPad, La Jolla, CA, USA).

## 3. Results

### 3.1. HNE Organoid Morphology Reflects That of Airway Epithelial Layers In Vivo

**Figure 1:**
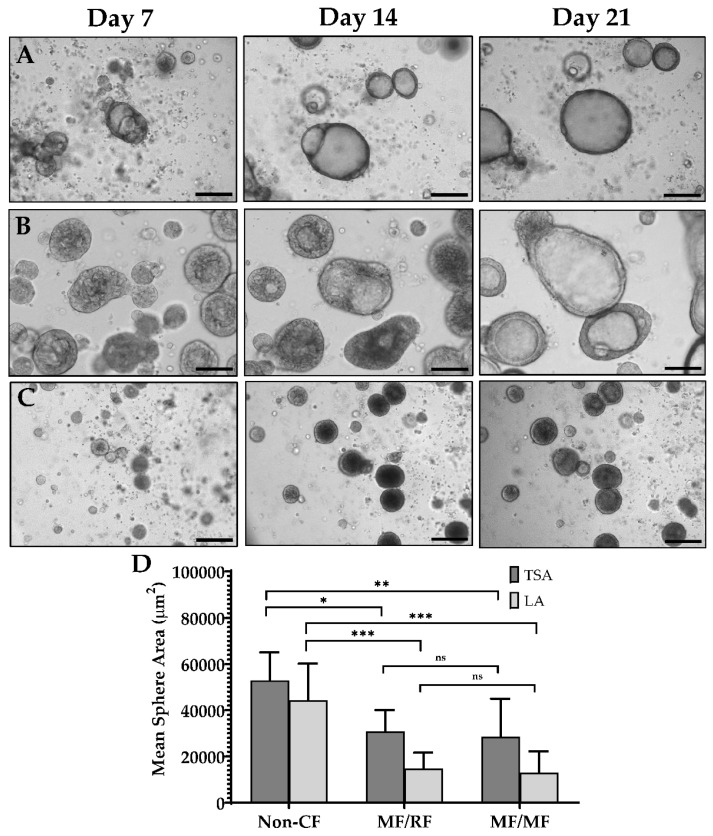
Nasal organoid growth characteristics. (**A**–**C**) Light microscopy of organoid formation over three weeks. (**A**) Non-CF (**B**) *G551D/Unknown* (MF/RF) (**C**) *F508del/F508del* (MF/MF). (**D**) The mean total surface area (TSA) and mean luminal area (LA) at 21 days of culture for non-CF (n = 6), MF/RF (n = 5), and MF/MF (n = 11) subjects’ organoids. MF = Minimal Function; RF = Residual Function. Scale = 250 µm. * *p* = 0.02, ** *p* = 0.005, *** *p* = 0.0001, ns = not significant.

**Figure 2:**
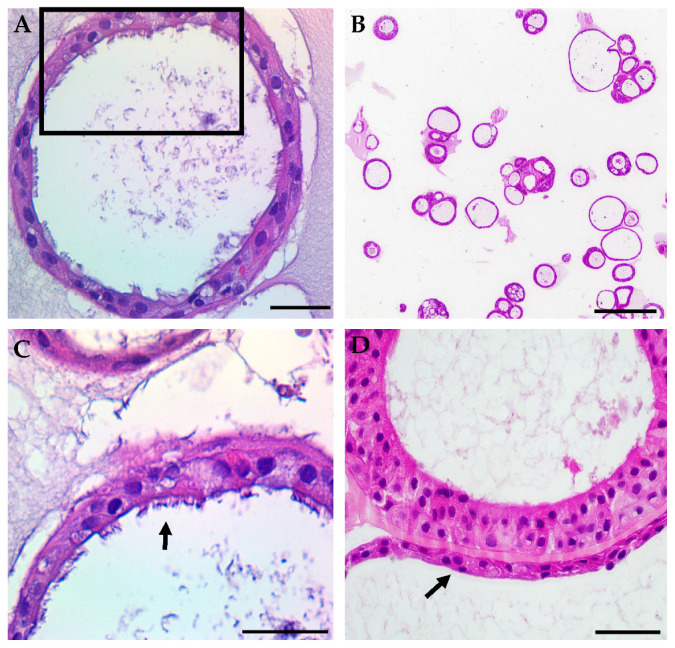
Representative samples of typical hematoxylin and eosin staining of HNE organoids show structural markers of differentiation. (**A**) Fully differentiated cross-section of a non-CF organoid shows a differentiated monolayer with an open lumen. Scale = 50 µm. (**B**) An example of the lower magnification of non-CF organoids demonstrates variations in the overall organoid morphology. Scale = 250 µm. (**C**) Higher magnification of the black frame in panel (**A**) showing a ciliated apical surface (arrow). Scale = 50 µm. (**D**) An example of higher magnification of G551D/unknown CF organoids showing thick and thin (arrow) epithelial walls. Scale = 50 µm.

Initial optimization of the assay included iterative testing of the Matrigel concentration, culture medium, cell seeding density, and type of culture vessel. After initial expansion of the nasal epithelial cells, we obtained organoids suitable for further testing from 88% of the successfully expanded samples. There were no significant differences in the culture success between CF and non-CF samples, similar to prior reports [20,21,22]. The average number of organoids formed in each well with the seeding density of 500 cells/µL was 31 (non-CF) and 37 (CF). Lumens were visible as early as day 3 of culture, and in the majority of samples by day 7 (Figure 1A–C). By 21 days of culture, the total surface area (TSA) and luminal area (LA) were significantly different between non-CF and CF patients, with two minimal function (MF) mutations or with a residual function (RF) mutation, but not between those with RF and MF (Figure 1D). Fixed cross-sections of the organoids show a robust circular culture with the apical lumen on the interior, and thick and thin epithelial walls, with evidence of cilia forming cells (Figure 2). Other than the lumen size, there were no qualitative differences observed between non-CF and CF cultures up to 42 days of weekly imaging.

**Figure 3:**
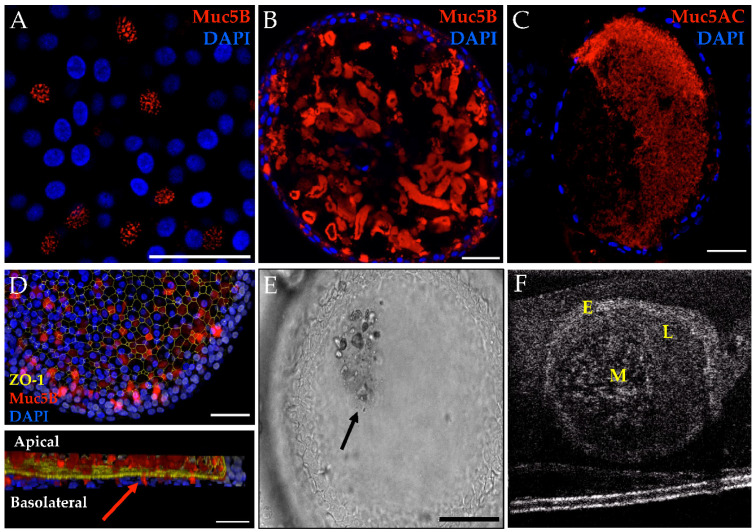
Mucus characteristics in HNE organoids, representative findings from CF and non-CF organoids are shown. (**A**) MUC5B granules in G551D/N1303K organoid. Scale = 50 µm. (**B**) Muc5B whole-mount IF showing a bundled mucin pattern in F508del/R117H-5T organoid. Scale = 50 µm. (**C**) MUC5AC fixed cross-section showing a diffuse mucin pattern in a non-CF organoid. Scale = 50 µm. (**D**) Top panel is the maximum projection of the 3-D hemisphere in the bottom panel. MUC5B is seen entering the lumen (arrow) through the apical surface in a non-CF organoid. Scale = 500 µm. (**E**) Brightfield microscopy showing a clump of cellular debris and/or mucus in the lumen in a non-CF organoid. Scale = 100 µm. (**F**) µOCT still of probable mucus in a non-CF organoid. (**E**,**F**) correspond to Videos **S1** and **S2**. E = epithelium L = lumen M = mucus.

Mucus expression was also assessed after 28 days of culture (Figure 3). Immunofluorescent staining of both MUC5AC and MUC5B was performed to assess the mucins produced, with similar qualitative expression regardless of the genotype. Mucus-producing cells with evidence of mucin granules were detected (Figure 3A). MUC5B shows bundles of mucins in both non-CF and CF organoids, whereas MUC5AC organizes as a diffuse web (Figure 3B,C). Although similar patterns have been seen in other published works, the differences in mucins seen may be due to the differences in the immunofluorescent techniques or CF phenotype [10,33]; while we did not observe the finding in a CF organoid, the approach did not allow for quantitative comparisons. Three-dimensional (3D) reconstruction of the confocal image demonstrates the secretion of MUC5B from mucosal cells into the lumen of the organoid (Figure 3D). Light microscopy and µOCT imaging were completed to assess luminal mucus movement (Figure 3E,F). Corresponding videos show a circular pattern of movement of the fluid and mucus within the lumen (Videos S1 and S2).

**Figure 4:**
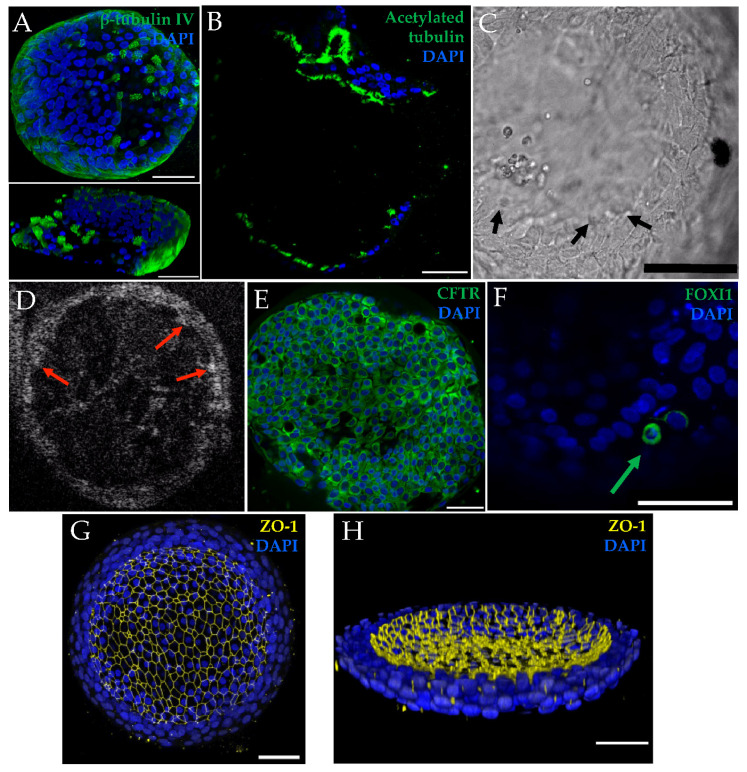
Other markers of airway epithelial differentiation are shown in selected images from non-CF and CF organoids. (**A**) Top panel is a confocal maximal projection of β-tubulin IV in F508del/G551D organoid with a 3-D reconstruction in the bottom panel that was sliced into to show cilia protruding. Scale = 50 µm. (**B**) Cross-section stained with acetylated tubulin in a non-CF organoid. Scale = 50 µm. (**C**) Light microscopy image of still cilia (arrows) in a non-CF organoid. Scale = 100 µm. (**D**) µOCT image of still cilia (arrows) in a non-CF organoid. (**C**,**D**) Stills correspond with Videos **S3** and **S4**. (**E**) Organoids also expressed CFTR, as seen in this representative example from F508del/R117H-5T organoids; co-localizion with ZO-1, seen in a non-CF organoid, is shown in Figure **S2**. Scale = 50 µm. (**F**) FOXI1-positive cell (arrow) in an F508del/F508del organoid. Scale = 50 µm. Maximal projection of tight-junction (ZO-1) staining (**G**) and a three-dimensional hemisphere demonstrating apical (luminal) localization in F508del/F508del organoids (**H**). Scale = 50 µm.

For both non-CF and CF organoids, cilia expression was assessed in histology sections (Figure 2A,C), whole-mount immunofluorescence with 3-D reconstruction (Figure 4A), immunofluorescent fixed cross-sections (Figure 4B), light microscopy (Figure 4C), and µOCT imaging (Figure 4D). Videos show cilia beating on the luminal (apical) surface of the organoid (Videos S3 and S4), and the ciliary beat frequency was readily measured (Figure S1). Cilia formation was similar across genotypes. Other markers of differentiation were also assessed with immunofluorescent staining of whole-mount fixed organoids. Apically localized tight junctions (ZO-1), CFTR, and FOXI1 (marker for ionocytes) were identified (Figure 4E–H). ZO-1 staining was similar across genotypes. The detection of CFTR was seen in both non-CF and CF cultures (MF/MF and MF/RF), similar to prior reports [34]. Apical plasma membrane localization of CFTR was presumed after co-staining with ZO-1 was visualized in a non-CF organoid (Figure S2) as previously seen in monolayers [35].

### 3.2. Organoid Lumen Size Differs between Different CFTR Genotypes

**Figure 5:**
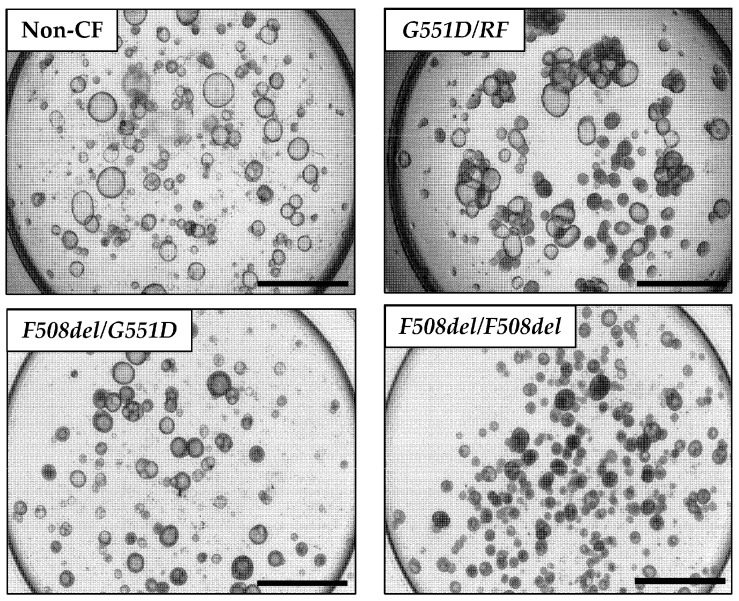
Light microscopy of cultures at 21 days of growth demonstrates variations in the amount of luminal fluid present within organoids of different CFTR genotypes. Scale = 1000 µm. The genotype (*G551D/unknown (MF/RF based on phenotype*) is defined in Supplementary Material Table S1.

**Figure 6:**
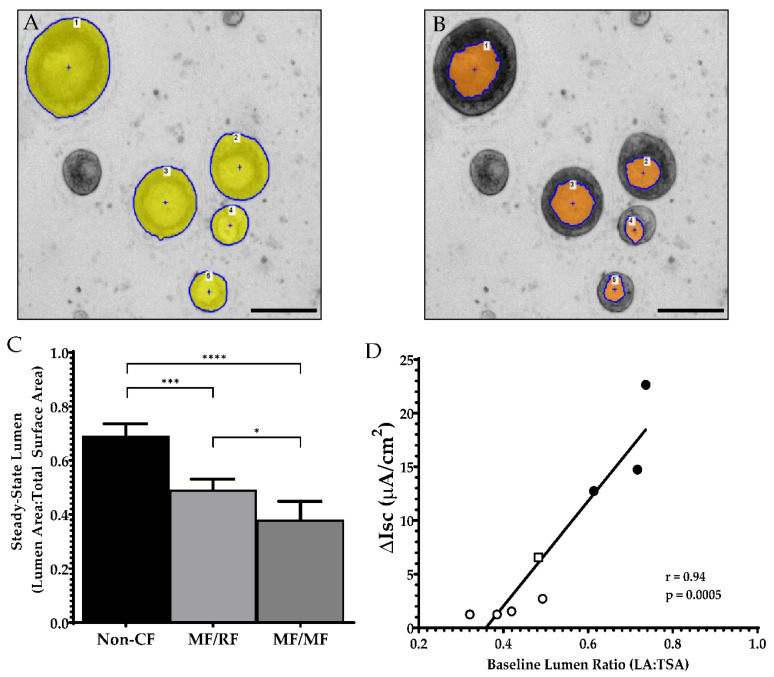
Baseline lumen ratio. Zoomed-in section of a well to show *F508del/G551D* organoids (**A**) masking of the organoid’s total surface area and (**B**) masking of the same organoids’ luminal areas. (**C**) Baseline lumen to total surface area ratio comparing non-CF (n = 6) to CF patients with residual function (n = 5) and minimal function mutations (n = 11). (**D**) Pearson correlation comparing the average organoid BLR for each person and the corresponding change in short-circuit current (∆Isc) after forskolin stimulation in the ussing chamber. Not all subjects from (**C**) had corresponding short-circuit current measurements. Filled circle = Non-CF; Open square = MF/RF; Open circle = MF/MF. Scale = 200 µm. * *p* = 0.02, *** *p* = 0.0001, and **** *p* < 0.0001.

By day 21, organoids were determined to be sufficiently differentiated for functional evaluation. At this time point, organoids exhibited a more uniform response than younger or older cultures. Assessment of the differences between non-CF and CF organoids showed striking contrasts. In Figure 5, representative images of 21-day-old cultures of non-CF organoids are compared to cultures from patients with CF (G551D/Unknown (residual function); F508del/G551D; and F508del/F508del). While TSA and LA can broadly distinguish between non-CF and CF phenotypes (Figure 1D), the baseline luminal ratio (BLR), the fraction of LA to TSA, can distinguish between non-CF and CF and between varying levels of CFTR dysfunction (Figure 6A–C). Furthermore, BLR shows a high correlation (*r* = 0.94, *p* = 0.0005) with the baseline forskolin-stimulated Isc in HNE monolayers from the same patients (Figure 6D).

### 3.3. Optimization of Forskolin-Induced CFTR-Dependent Swelling Assay

**Figure 7:**
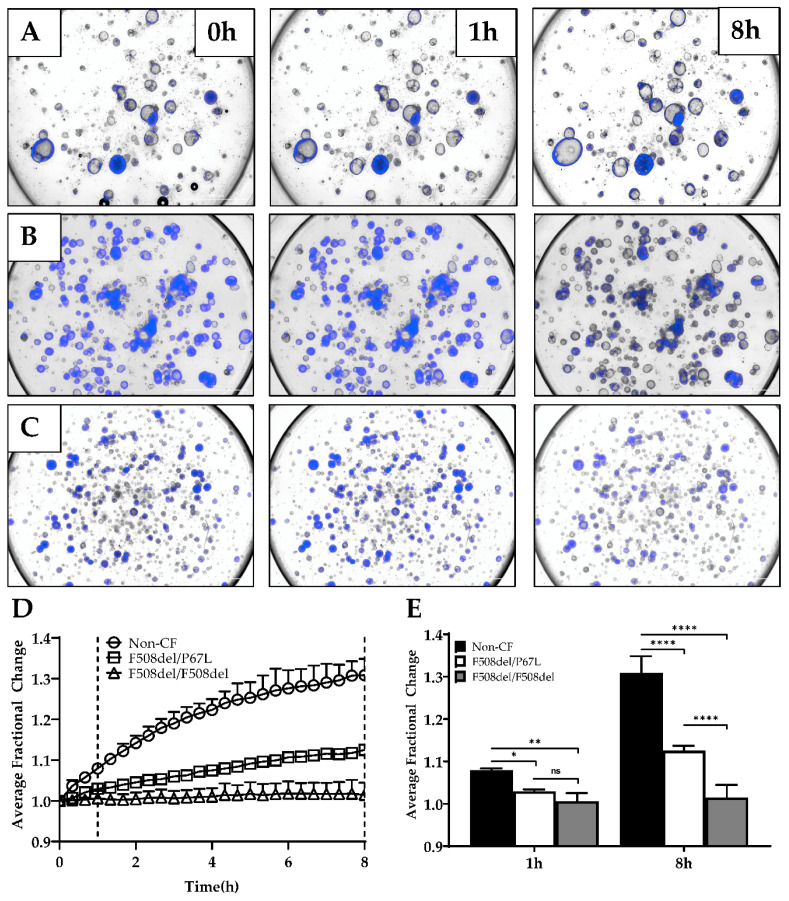
FIS assay. (**A**–**C**) Three representative brightfield and DAPI-stained images at times 0, 1, and 8 h for non-CF (**A**), F508del/P67L (**B**), and F508del/F508del (**C**) CFTR HNE organoids. (**D**) Average fractional change from baseline over 8 h for each subject, dotted lines highlighting the 1- and 8-h timepoints. (**E**) 1-h vs. 8-h time points for the average fractional change of non-CF, F508del/P67L, and F508del/F508del organoids (n = 1). (**A**) Corresponds with Video **S5**, (**B**) corresponds with Video **S6**, and (**C**) corresponds with Video **S7**. ns = not significant; * *p* = 0.0441, ** *p* = 0.0023, and **** *p* < 0.0001.

Organoid cultures have previously been shown to be predictors of CFTR-dependent fluid transport, including to test downstream effects of gene editing [10,29,36,37]. These assays use short (1–2 h) time-lapse imaging [10,29]. We tested this organoid model for applicability for forskolin-induced swelling (FIS). We hypothesized that longer assays might show a greater dynamic range since fluid transport in epithelia is a complex process and may not be evident in a short time [11]. HNE organoids derived from cells from a healthy non-CF patient (Figure 7A), a patient with F508del/P67L (Figure 7B), and a patient with F508del/F508del (Figure 7C) CFTR are shown at 0, 1, and 8 h (Videos S5–S7). Figure 7D is the average fractional change over 8 h for three subjects with varying levels of baseline CFTR function (non-CF = normal function; F508del/P67L = residual function; and F508del/F508del=minimal function). In Figure 7E, the fractional change for each subject is shown, showing significant distinctions for the CFTR function between all three subjects at 8 h while not at 1 h.

## 4. Discussion and Conclusions

In this manuscript, we provide evidence for a highly differentiated organoid culture that expresses markers consistent with human airway epithelial monolayers with reproducible cultures regardless of the genotype. The organoid culture shows distinct differences in lumen formation between non-CF and CF cultures with and without residual CFTR function, suggesting a CFTR dependence of the lumen size and fluid transport during organoid development. Along with the significant differences between the lumen formation of CF and non-CF organoids, this difference highly correlates with paralleled short-circuit measurements of baseline CFTR activity. This has not previously been evaluated in human nasal epithelial organoids, but a similar finding was seen in intestinal organoids [38]. Our swelling data suggest that this model has the potential for functional assessment of the patient phenotype, identifying both large and small differences (such as residual function, or a modest improvement of one treatment over another). Furthermore, the model has multiple applications, including FIS, assessment of mucus expression, transport, cilia activity, and fluorescence microscopy.

We present data that the full effects of CFTR restoration may not be visible over short time frames in nasal organoid studies. By lengthening the assay to eight hours, we were able to increase the distinction between differing CFTR activity levels. This result requires further testing with more subjects, but it is an interesting finding that takes into account that fluid transport may not immediately follow CFTR rescue, and the complex systems that result in fluid secretion into the lumen may take some time to show full effects. The functional assay could be useful in pre-clinical testing, where interventions designed to be delivered via systemic absorption (i.e., from the basolateral side after distribution through the blood) can be applied to an in vitro surrogate. Because it requires so little material to generate many replicates, it is amenable to higher throughput applications using novel compounds or iterative testing of different delivery mechanisms for RNA- and DNA-based therapies. Additionally, it provides a useful ex vivo marker for in vivo gene therapy trials. A nasal brush biopsy is safe, easily obtained in an outpatient clinical research setting, and can be repeatedly collected from the patient [39]. This could be tested to monitor the stability of a genetic repair, which would be helpful to confirm a corrected phenotype even if clinical measures (like lung function testing) may not be altered (for example, if a patient has a normal lung function result at the start of the study).

The differentiation of this model provides multiple readouts for CFTR functional rescue. It is highly amenable to fluorescent imaging. We used fluorescence for automated imaging of living cells as well as fixed whole organoids for immunofluorescent staining of specific markers. We observed limited evidence that the organoid can recapitulate mucin organization also seen in other models [33]. Currently, a variety of approaches to deliver gene therapy packages exist, and delivery mechanisms tagged with a fluorescent marker for measuring uptake could easily be tested across a variety of patient samples with this approach. Further, the readout for functional assessment of CFTR rescue is not limited to the measurement of the total cross-sectional area or lumen over time. Our results show that µOCT imaging can yield multiple readouts of the lumen area, mucus morphology, ciliary function, and possibly with further testing, mucus viscosity and mucociliary transport speeds.

Our study has some limitations. We present functional data (lumen size and swelling) from a limited number of subjects, while additional subject samples were used for iterative testing to optimize the culturing and assay methodology to achieve a reproducible assay. Despite the small number of subjects, the lumen measurement shows excellent distinction for differences between patient groups. The current analysis limits our baseline luminal ratio assay to manual measurements until further revisions to the automated software can be made. However, because the measurements and output for the FIS assay are automated, we will now be able to scale up the testing of many patients and correlate clinical outcome measures with the organoid data for future studies.

Our results show that a solid assay with similarities to the robust intestinal organoid forskolin-induced swelling assay can be developed with HNEs. This provides a significant advantage because not every CF center has the capacity to collect rectal biopsies on all patients, nor are all patients receptive to biopsy collection. However, many CF centers in the United States and elsewhere have experience in collecting nasal brush biopsies from infants through to adults, with risk comparable to that of a nasopharyngeal swab for virus detection. The model can assess CFTR dysfunction and rescue, both as a pre-clinical tool for systemically delivered DNA-based therapies and an ex vivo surrogate biomarker to assess the stability of DNA-based therapy in clinical trials.

## Supplementary Materials

The following are available online at https://www.mdpi.com/2073-4425/11/6/603/s1, Table S1: Individual subject demographics and available clinical data for selected participants contributing functional data. Figure S1: Example measurement of µOCT ciliary beat frequencies, Figure S2: Organoid CFTR (green) co-localization with apical ZO-1 (red). (A) To illustrate the location of the ZO-1 staining within the organoid, an α-blended 3-D volume reconstruction was created with the fluorescence intensity increased to reveal the organoid’s shape and orient the reader to the location of the 2D co-localization image below. (B) Magnified view of the yellow frame in (A) to better visualize the CFTR and ZO-1 overlay, Video S1: Brightfield video of luminal movement of mucus, Video S2: µOCT video of luminal movement of mucus, Video S3: Brightfield video of luminal cilia beating, Video S4: µOCT video of cilia beating, Video S5: FIS assay of non-CF organoids, Video S6: FIS assay of F508del/P67L organoids. Video S7: FIS assay of F508del/F508del organoids.

## Abbreviations

CF	cystic fibrosis
CFTR	Cystic Fibrosis Transmembrane conductance regulator
