# Supplementary material for: Human Nasal Epithelial Organoids for Therapeutic Development in Cystic Fibrosis
Source: Genes (Basel). 2020 May 29;11(6):603. doi: 10.3390/genes11060603 (PMC7349680; doi:10.3390/genes11060603)
Supplement: Supplementary file 1 [file genes-11-00603-s001.zip › Supplementary Material_v2/Table S1.docx]

| **Subject** | **Age** | **Sex** | **Genotype** | **Sweat Chloride**  **(mmol/L)** | **CF Phenotype** |
| --- | --- | --- | --- | --- | --- |
| A | 23 | F | F508del/G551D | 110 | MF |
| B | 44 | M | F508del/P67L | 56 | RF |
| C | 17 | M | F508del/F508del | -- | MF |
| D | 51 | F | G551D/Unknown* | 41 | RF |
| E | 17 | F | F508del/G551D | 107 | MF |
| F | 24 | F | F508del/G551D | 116 | MF |
| G | 24 | M | F508del/F508del | -- | MF |
| H | 16 | F | F508del/F508del | 116 | MF |
| I | 17 | F | F508del/F508del | 114 | MF |
| J | 19 | F | F508del/N1303K | 105 | MF |
| K | 1 | F | G551D/S108F-5T-TG12 | 86 | RF |
| L | 47 | F | F508del/R117H-5T | -- | RF |
| M | 43 | M | I507del/R117H-5T | -- | RF |
| N | 10 | F | F508del/F508del | 85 | MF |
| O | 29 | F | F508del/G551D | 105 | MF |
| P | 17 | M | F508del/G551D | 104 | MF |

MF= minimal function; RF= residual function; Clinical data for some participants was not available.

*Patient diagnosed by the CF clinical team on the basis of bronchiectasis, chronic sinusitis, reduced lung function, chronic *P. aeruginosa* infection of the lungs requiring hospitalization and recurrent intravenous antibiotic treatment; non-smoker status; family history of CF; and evidence of CFTR dysfunction shown by indeterminate sweat chloride and nasal potential difference measurements. This patient is pancreatic sufficient. The patient was genotyped using methodology that is not currently considered comprehensive and has not been repeated by the clinical team since the patient otherwise meets criteria for diagnosis and other diagnoses were excluded. We conclude that the second mutation has not been identified, and label it as “unknown”.
