# Supplementary figures and images for: Human Nasal Epithelial Organoids for Therapeutic Development in Cystic Fibrosis
Source: Genes (Basel). 2020 May 29;11(6):603. doi: 10.3390/genes11060603 (PMC7349680; doi:10.3390/genes11060603)

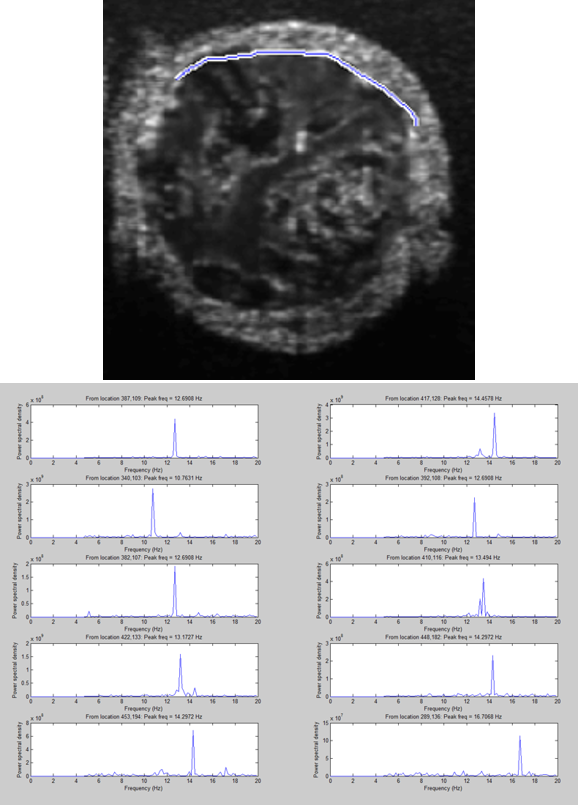

Supplement: Supplementary file 1 [file genes-11-00603-s001.zip › Supplementary Material_v2/Figure S1.tif]

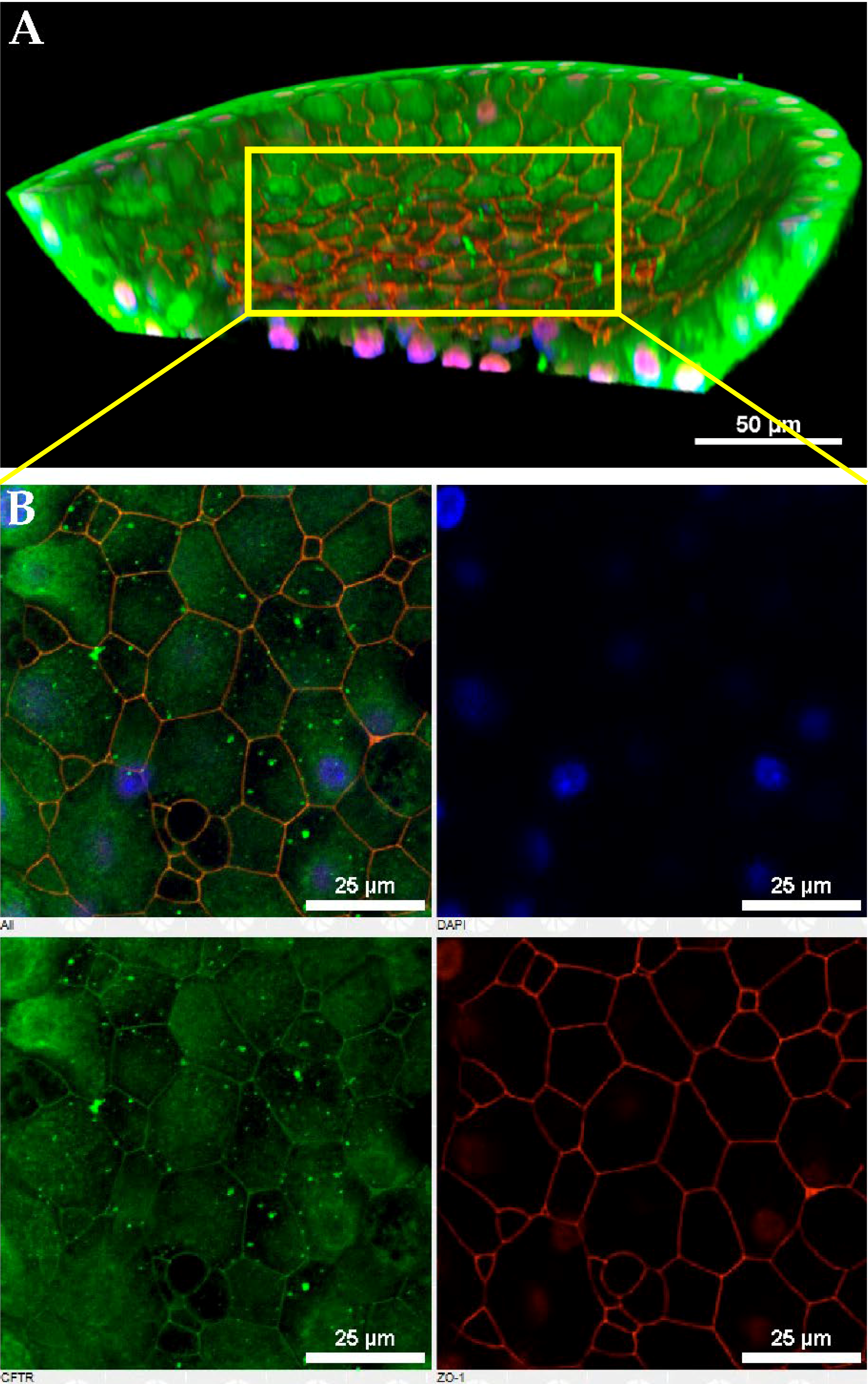

Supplement: Supplementary file 1 [file genes-11-00603-s001.zip › Supplementary Material_v2/Figure S2.tif]
